# Chaihuang Qingyi Huoxue granule ameliorates severe acute pancreatitis by modulating gut microbiota and repairing the intestinal mucosal barrier

**DOI:** 10.3389/fcimb.2025.1514201

**Published:** 2025-02-18

**Authors:** Xiaobin Zhang, Xusen Zeng, Wen Guo, Xin Zhou, Yi Zhang, Mingyun Tang, Juan Fu, Yuqing Deng, Xin Liang, Long Zhao, Zhi Li, Tiangang Wang, Li Li, Guohui Xiao

**Affiliations:** ^1^ School of Integrated Traditional Chinese and Western Medicine, Southwest Medical University, Luzhou, Sichuan, China; ^2^ Department of Spleen and Stomach Diseases, The Affiliated Traditional Chinese Medicine Hospital, Southwest Medical University, Luzhou, Sichuan, China; ^3^ The Key Laboratory of Integrated Traditional Chinese and Western Medicine for Prevention and Treatment of Digestive System Diseases of Luzhou city, The Affiliated Traditional Chinese Medicine Hospital, Southwest Medical University, Luzhou, Sichuan, China; ^4^ Department of Gastroenterological Surgery, Leshan Hospital of Traditional Chinese Medicine, Leshan, Sichuan, China; ^5^ School of Integrated Traditional Chinese and Western Clinical Medicine, North Sichuan Medical College, Nanchong, Sichuan, China

**Keywords:** severe acute pancreatitis, traditional Chinese medicine, Chaihuang Qingyi Huoxue Granule, gut microbiota, intestinal mucosal barrier, short-chain fatty acids

## Abstract

**Background:**

During severe acute pancreatitis (SAP), damage to the intestinal mucosal barrier and translocation of intestinal pathogenic bacteria are key mechanisms that accelerate the disease progression of SAP. Chaihuang Qingyi Huoxue Granule (CH) is a herbal formula used in the clinical treatment of SAP. This study aims to investigate the role of CH in regulating gut microbiota and intestinal mucosal barrier in SAP rats.

**Methods:**

Sodium taurocholate (3.5%) was retrogradely perfused into the biliopancreatic duct to establish the model of SAP in rats. CH (4.4 g/kg) was administered by gavage. Serum amylase, lipase, and endotoxin levels were measured. Hematoxylin-eosin (HE) staining was used to observe morphological changes in the pancreas and colon. The expression of zona occludens-1 (ZO-1) and occludin in the colon was examined by immunohistochemistry (IHC) and western blot. 16S rDNA gene sequencing was used to analyze the gut microbiota of the rats. The content of short-chain fatty acids (SCFAs) in the intestinal contents of the rats was determined by gas chromatography-mass spectrometry (GC-MS).

**Results:**

CH reduced serum amylase, lipase, and endotoxin levels in SAP rats, alleviated pathological damage in the pancreas and colon, and restored the expression of ZO-1 and occludin. Moreover, CH alleviated gut microbiota dysbiosis in SAP rats, with restored gut microbiota diversity and structure. At the phylum level, the relative abundance of Firmicutes and Bacteroidetes increased, while that of Proteobacteria decreased. At the genus level, the abundance of Ruminococcus 1, Parabacteroides, Prevotellaceae UCG-001, Lachnospiraceae NK4A136 group, and Lactobacillus increased, while that of Escherichia-Shigella, Enterococcus, and Enterobacter decreased. In addition, CH increased the levels of SCFAs in the intestinal contents of SAP rats.

**Conclusion:**

CH ameliorates SAP by maintaining the homeostasis and diversity of the gut microbiota, increasing the levels of SCFAs, and repairing the intestinal mucosal barrier.

## 1 Introduction

Acute pancreatitis (AP) is a common acute abdominal condition in which severe acute pancreatitis (SAP) is characterized by persistent multiple organ failure with up to 30% mortality (Dai et al., 2021). The mechanisms of intestinal barrier dysfunction induced by SAP are thought to involve inflammatory response, oxidative stress, endocrine disorders, and mitochondrial injury (Liu et al., 2022). SAP-associated intestinal mucosal barrier dysfunction can increase intestinal permeability, leading to bacterial and endotoxin translocation into the systemic circulation, triggering an inflammatory cascade and eventually leading to systemic inflammatory response syndrome (SIRS) and multiple organ dysfunction syndrome (MODS) (Ma et al., 2021). Due to microcirculatory dysfunction, hypovolemia, splanchnic vasoconstriction, and ischemia-reperfusion injury (Wu et al., 2014), the gut acts as the starter for severe systemic complications in SAP. Therefore, one of the main goals of treatment in the early phases of SAP should be to maintain the integrity of the intestinal barrier (Capurso et al., 2012).

In addition to viruses, fungi, archaea and protozoa, bacteria are the predominant hosts in the human intestine (Abenavoli et al., 2022). The gut microbiota is involved in nutrient absorption, modulation of intestinal permeability, and the construction of intestinal barriers (Lu et al., 2021), which is pivotal in the human immune system and intestinal homeostasis (Parker et al., 2022). There are significant changes in the composition and relative abundance of the gut microbiota during SAP, with studies showing an increase in the relative abundance of Proteobacteria and Actinobacteria at the phylum level and a decrease in Firmicutes and Bacteroidetes. At the genus level, the abundance of facultative anaerobes such as Escherichia-Shigella, Enterococcus, and Enterobacter increased, while beneficial bacteria such as Ruminococcaceae, Prevotellaceae UCG-001, Lactobacillus, and Parabacteroides decreased (Zhu et al., 2021; Wang et al., 2022, 2023). The intestinal mechanical barrier is a crucial component of the mucosal barrier, consisting of intestinal epithelial cells (IECs) and tight junctions (TJs). Regulating the abundance of beneficial gut microbiota can promote the expression of TJ proteins such as zona occludens-1 (ZO-1) and occludin to maintain the integrity and stability of the intestinal mucosal barrier and prevent disease progression (Wang et al., 2019; Mo et al., 2022). Short-chain fatty acids (SCFAs), which serve as the primary energy source for IECs, increase the expression of ZO-1 and occludin proteins, thereby mitigating the intestinal mucosal barrier damage (Huang et al., 2021). Therefore, maintaining gut microbiota homeostasis and repairing the intestinal mucosal barrier are considered therapeutic strategies to prevent and alleviate SAP.

Chaihuang Qingyi Huoxue Granule (CH) is a herbal formula based on the traditional Chinese medicine (TCM) theory of “unblocking and descending”. It consists of Bupleurum chinensis DC. (Chaihu), Cortex Magnoliae Officinalis (Houpu), Radix Paeoniae Rubra (Chishao), Rheum palmatum (Dahuang), Persicae Semen (Taoren), Salviae miltiorrhiza (Danshen), Radix Glycyrrhizae (Gancao), Rhizoma Corydalis (Yanhusuo), Astragali Radix (Huangqi), Scutellariae Radix (Huangqin), Fructus Aurantii Immaturus (Zhishi), Gardenia jasminoides Ellis (Zhizi), Paeoniae Radix Alba (Baishao), Taraxacum mongolicum Hand. - Mazz. (Pugongying). CH has been confirmed to alleviate symptoms and improve clinical efficacy in SAP patients (Shi et al., 2019). Previous studies have demonstrated that CH can alleviate SAP by inhibiting the inflammatory response and oxidative stress (Li et al., 2023; Yang et al., 2024b), and it has been found that CH can regulate the PI3K/AKT pathway and reduce the expression of inflammatory mediators such as interleukin-6 (IL-6), interleukin-1β (IL-1β), tumor necrosis factor-α (TNF-α) to alleviate SAP (Yang et al., 2024a). However, whether CH can alleviate SAP by mediating the gut microbiota and its metabolites in SAP is unclear. Therefore, by establishing SAP rat models, this study explored the potential mechanism of CH in alleviating the symptoms of SAP rats through the gut microbiota, SCFAs, and intestinal mucosal barrier.

## 2 Materials and methods

### 2.1 Materials

CH (Lot: 20220721) was purchased from the Affiliated Traditional Chinese Medicine Hospital of Southwest Medical University. The extraction methods and quality control were described in detail in a previous study (Zhao et al., 2021). Sodium taurocholate (T9034) was purchased from Sigma (New Mexico, USA). Sodium pentobarbital (170108) was purchased from Red Rock Reagent Factory (Tianjin, China). HE detection kit (C0105S) was purchased from Beyotime (Shanghai, China). The quantitative end-point chromogenic tachypieus amebocyte lysate assay kit (EC80545S) was purchased from Xiamen Houshiji (Xiamen, China). ZO-1 antibody (21773-1-AP) and occludin antibody (66378-1-lg) were purchased from Proteintech (Wuhan, China). High-efficiency RIPA lysis buffer (PC101), Bicinchoninic Acid Assay (BCA) kit (ZJ102), and high-sensitivity Electrochemiluminescence (ECL) detection kit (SQ201) were purchased from Epizyme (Shanghai, China). GAPDH antibody (AF7021) and horseradish peroxidase (HRP)-conjugated secondary antibody (S0001) were purchased from Affinity (Jangsu, China).

### 2.2 Animal studies

48 SPF male Sprague-Dawley rats (250 ± 20 g) were obtained from the Animal Experimental Research Centre of Southwest Medical University and housed under pathogen-free conditions (a regular 12/12 h light/dark cycle, 22 ± 2°C temperature, and 40-70% relative humidity) throughout the experiment. All experiments were approved by the Animal Ethics Committee of Southwest Medical University (No. 20231016-014). All rats had free access to standard rodent chow and water and were acclimated to the environment for 1 week before the formal experiment, and then were randomly divided into a control group (SHAM group), a model group (SAP group), and an intervention group (CH group), with 16 rats in each group, and the groups were divided into two subgroups of 8 rats each, a 12h group and a 24h group, according to the time after administration by gavage (12 hours and 24 hours). Rats in the SAP and CH groups were induced as SAP models by retrograde injection of 3.5% sodium taurocholate into the biliopancreatic duct (Hu et al., 2022), and the SHAM group received the same surgical intervention except that they were given 0.9% saline instead. The CH group received CH (4.4 g/kg) by gavage 6 hours after modeling. The SHAM and SAP groups received equal amounts of saline by gavage. Rats were sacrificed after 12 and 24 hours. Blood, pancreas, colon, and fresh feces samples were collected for further analysis.

### 2.3 Histopathological examination and scoring

Fresh pancreas and colon tissues were promptly fixed in a 4% paraformaldehyde solution, then dehydrated and embedded in paraffin. The embedded samples were stained with hematoxylin-eosin (HE) staining solutions. Histopathological evaluation was performed under a light microscope in a double-blind approach with the Schmidt pathology scoring criteria (Schmidt et al., 1992) for pancreatic tissue and the Chiu pathology scoring criteria (Chiu et al., 1970) for colonic tissue.

### 2.4 Determination of serum amylase, lipase and endotoxin levels

Serum amylase and lipase levels were measured using a fully automated biochemical analyzer (Olympus, Japan). Serum endotoxin was determined by a quantitative end-point chromogenic tachypieus amebocyte lysate assay kit.

### 2.5 Immunohistochemistry (IHC) analysis

The paraffin-embedded sections of colon tissue were deparaffinized, rehydrated, and sealed with bovine serum albumin. The sections were successively incubated with primary antibodies of ZO-1 (1:200), occludin (1:200) and the corresponding secondary antibodies, stained with 3,3'-diaminobenzidine (DAB) and hematoxylin, and visualized under a light microscope (Leica, Germany). Finally, the results were analyzed using the Image J software (NIH, USA).

### 2.6 Western blot

Rats colon tissues were lysed in high-efficiency RIPA lysis buffer on ice. Isolated total intestine protein was quantified by the BCA detection method, 50 µg of protein per well was loaded for 7.5% SDS-PAGE gel electrophoresis and then transferred to a PVDF membrane. The membrane was blocked in 5% skim milk for 2 hours at room temperature and incubated with the specified primary antibodies (ZO-1, 1:5000 and occludin, 1:5000) at 4°C overnight, using GAPDH (1:20000) serving as an internal reference protein. The membrane was then incubated with HRP-conjugated secondary antibody (1:5000) at room temperature for 1 h. Signals were developed using the high-sensitivity ECL detection kit and images were captured using the TOUCH IMAGER™ (e-Blot, China). Image J software (NIH, USA) was used to analyze the grey intensity of the protein bands.

### 2.7 Microbial DNA extraction and 16S rDNA sequencing analysis

According to the manufacturer’s instructions, total microbial genomic DNA was extracted from all groups of fecal samples using the CTAB/SDS method and the purity determined on agarose gels. The V3-V4 region of the 16S rDNA gene was amplified with 341F-806R primers. The PCR products were purified using the AxyPrepDNA gel recovery kit (AXYGEN, USA). Sequencing libraries were generated using the NEB Next^®^Ultra™DNA Library Prep Kit for Illumina (NEB, USA) and sequenced on the Illumina NovaSeq 6000 platform. Library preparation and sequencing were conducted at Shanghai Zhongke New Life Biotechnology Co., Ltd. (Shanghai, China).

### 2.8 Microbiome bioinformatic analysis

Sequences with 97% identity were clustered into operational taxonomic units (OTUs) using Uparse software, and then diversity analysis, difference analysis, correlation analysis, and functional prediction analysis were performed. Alpha and beta diversity analyses were performed using the diversity plugin in the R language (R vegan package). Wilcoxon rank-sum test and ANOVA test (R stat package) were used to identify bacterial taxa with different abundances between groups at the phylum and genus level. Linear discriminant analysis (LDA) effect size (LEfSe) method was used to evaluate the influence of each differentially abundant taxon. Microbial function was predicted and analyzed by phylogenetic investigation of communities using reconstruction of unobserved states (PICRUSt) analysis based on Kyoto Encyclopedia of Genes and Genomes (KEGG) pathways (R ggpicrust2 package). LEfSe and PICRUSt analyses were performed in (https://www.bioincloud.tech/) (Gao et al., 2024). Spearman correlation analysis was used to assess the correlation between important bacterial taxa, SCFAs and disease phenotype.

### 2.9 SCFAs extraction and gas chromatography-mass spectrometry (GC-MS) analysis

Fecal samples were sequentially mixed with 0.5% phosphoric acid and ethyl acetate, centrifuged to obtain the supernatant, and 4-methylpentanoic acid was added as an internal control. GC-MS analysis was performed using a 7890 B gas chromatograph-5977 B mass spectrometer (Agilent, USA) with a capillary column (DB-FFAP, 30 m × 0.25 mm × 0.25 µm, Agilent, USA). The temperature was initially set at 90°C, then increased to 160°C at 10°C/min, and finally to 240°C at 40°C/min and held at this temperature for 5 min. The injector, ion source, quadrupole, and transfer line temperatures (°C) were 250, 230, 150, and 250, respectively. The helium carrier gas flow rate was maintained at 1 mL/min. MS conditions were electron impact ionization source, SCAN/SIM scan mode, and electron energy 70 eV.

### 2.10 Statistical analysis

Data were expressed as the mean ± standard error of mean (SEM), examined using SPSS 26.0 (SPSS Inc., USA), and performed with either R (version 4.3.3). P < 0.05 was considered to be statistically significant.

## 3 Results

### 3.1 CH alleviated pancreatic enzyme hyperactivation and pancreatic injury in SAP rats

**Figure 1:**
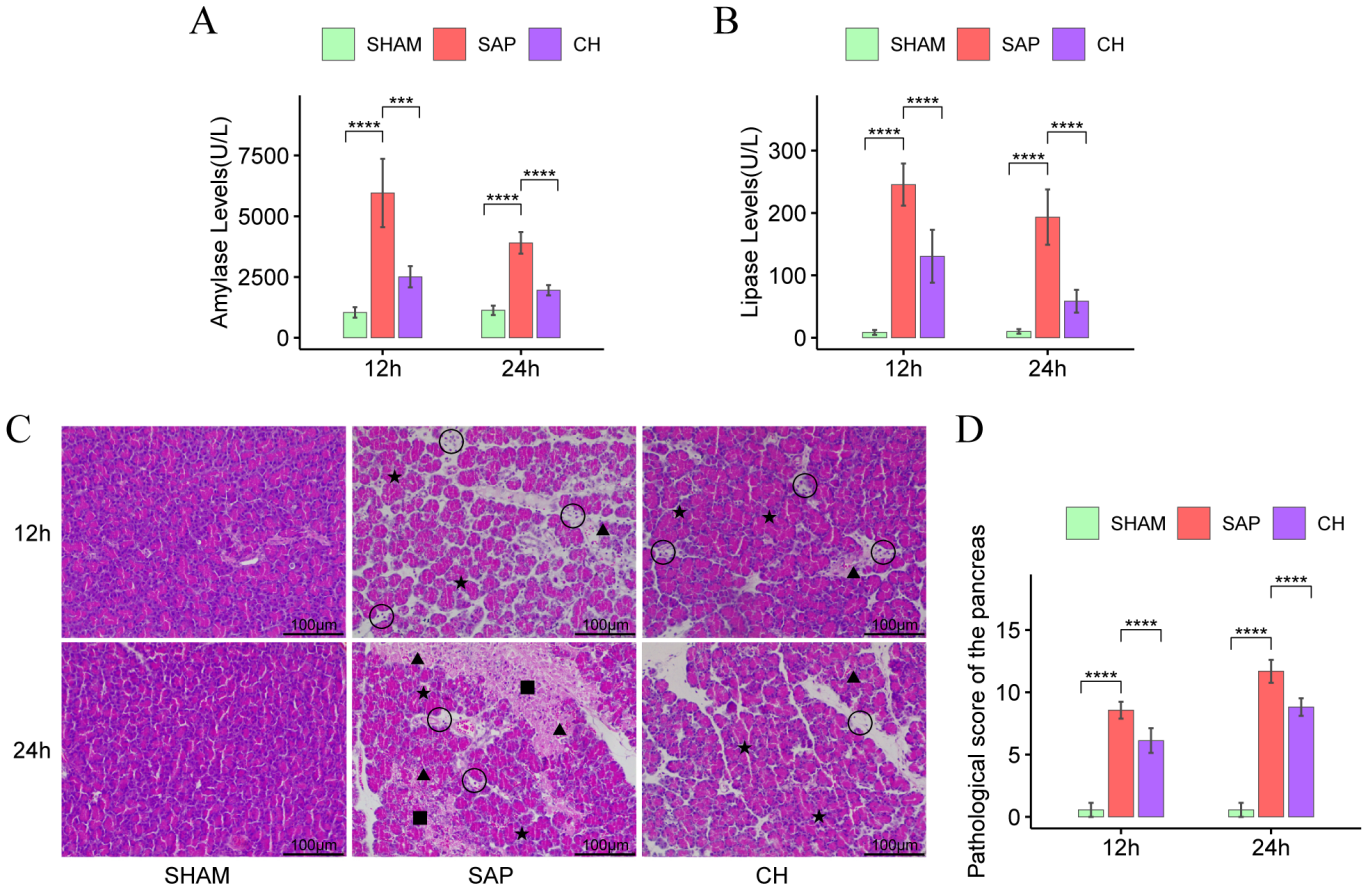
CH alleviated pancreatic enzyme hyperactivation and pancreatic injury in SAP rats. **(A)** The level of serum amylase. **(B)** The level of serum lipase. **(C)** Representative HE staining of the pancreas (× 200). Asterisks indicate edema, triangles indicate hemorrhage, squares indicate acinar necrosis, and circles indicate infiltrated immune cells. **(D)** Pathological damage score of the pancreas. (n = 8, ***P < 0.001, ****P < 0.0001, vs. SAP group).

Serum amylase and lipase are important markers in the diagnosis of SAP. In the same subgroups at 12h and 24h, both serum amylase (**Figure 1A**) and lipase (**Figure 1B**) levels were significantly higher in the SAP group compared to the SHAM group (P < 0.05). In contrast, serum amylase and lipase levels were substantially lower in the CH group compared to the SAP group (P < 0.05). In addition, according to the HE staining results (**Figure 1C**), in the same subgroups at 12h and 24h, the pancreatic tissue structure was significantly damaged in the SAP group compared to the SHAM group, with pancreatic acinar cell necrosis, interstitial edema, inflammatory cell infiltration, local vascular congestion, and significantly higher pathological scores (P < 0.05). In contrast, pancreatic pathological damage was significantly attenuated and pathological scores (**Figure 1D**) were significantly decreased in the CH group compared to the SAP group (P < 0.05). In conclusion, CH could alleviate pancreatic damage and thus alleviate the symptoms of SAP.

### 3.2 CH restored intestinal mucosal barrier function and reduced intestinal permeability in SAP rats

**Figure 2:**
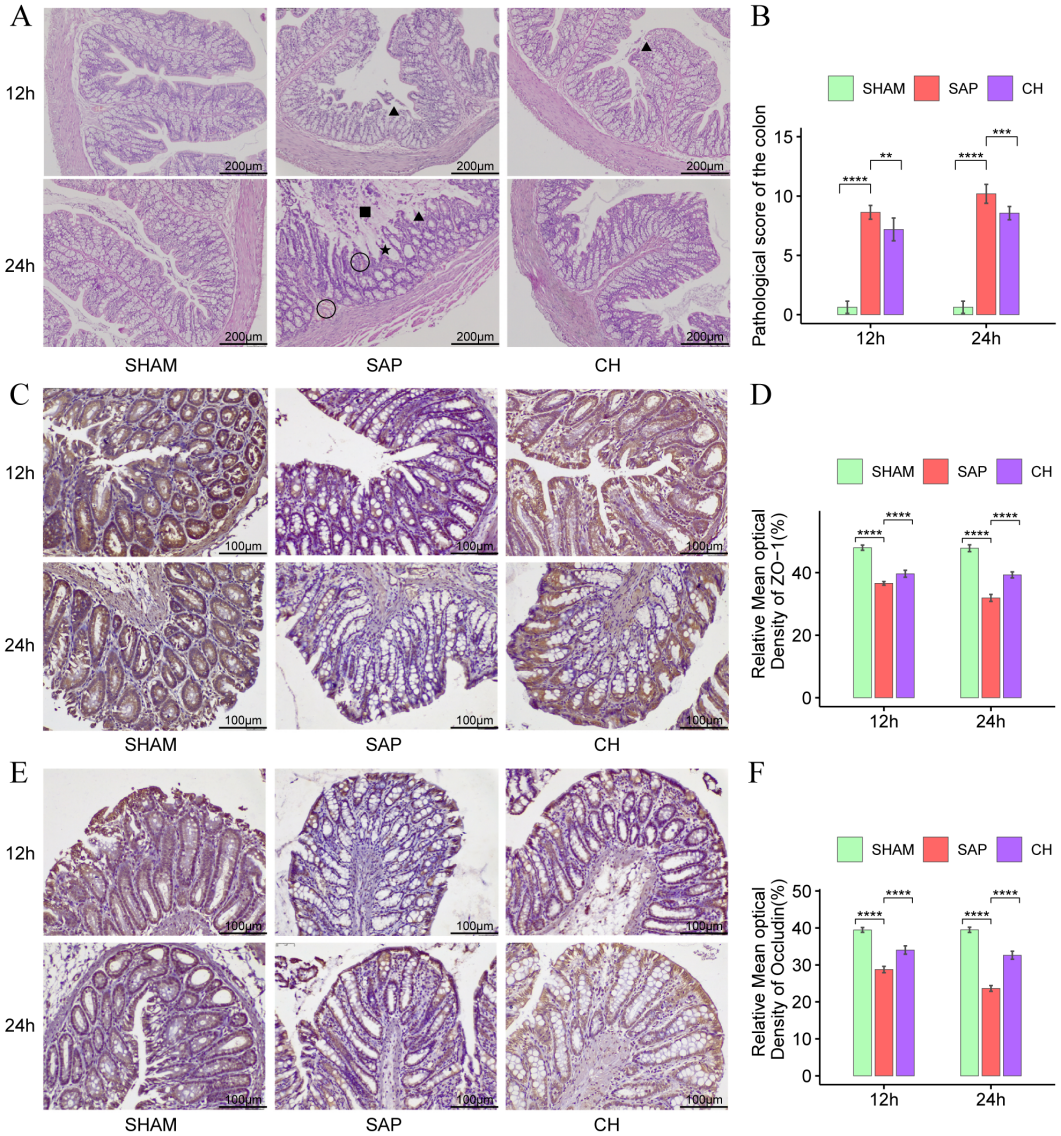
CH ameliorated intestinal damage and restored intestinal mucosal barrier function in SAP rats. **(A)** Representative HE staining of the colon (× 100). Asterisks indicate crypt distortion, triangles indicate epithelial shedding and denuded villi, squares indicate necrosis, and circles indicate hemorrhage. **(B)** Pathological damage score of the colon. **(C)** Immunohistochemical analysis of ZO-1 (× 200). **(D)** Expression of ZO-1 by immunohistochemistry. **(E)** Immunohistochemical analysis of occludin (× 200). **(F)** Expression of occludin by immunohistochemistry. (n = 8, **P < 0.01, ***P < 0.001, ****P < 0.0001, vs. SAP group).

According to the results of HE staining of colonic tissues (**Figure 2A**), in the same subgroups at 12h and 24h, the SAP group showed significant damage to the colonic mucosal tissue structure, absence of glands and crypts, necrosis of mucosal epithelial cells, detachment of intestinal villi, infiltration of inflammatory cells, and significantly higher colonic pathological scores (**Figure 2B**) compared to the SHAM group (P < 0.05). In contrast, the colonic pathological scores were significantly decreased in the CH group compared to the SAP group (P < 0.05).

**Figure 3:**
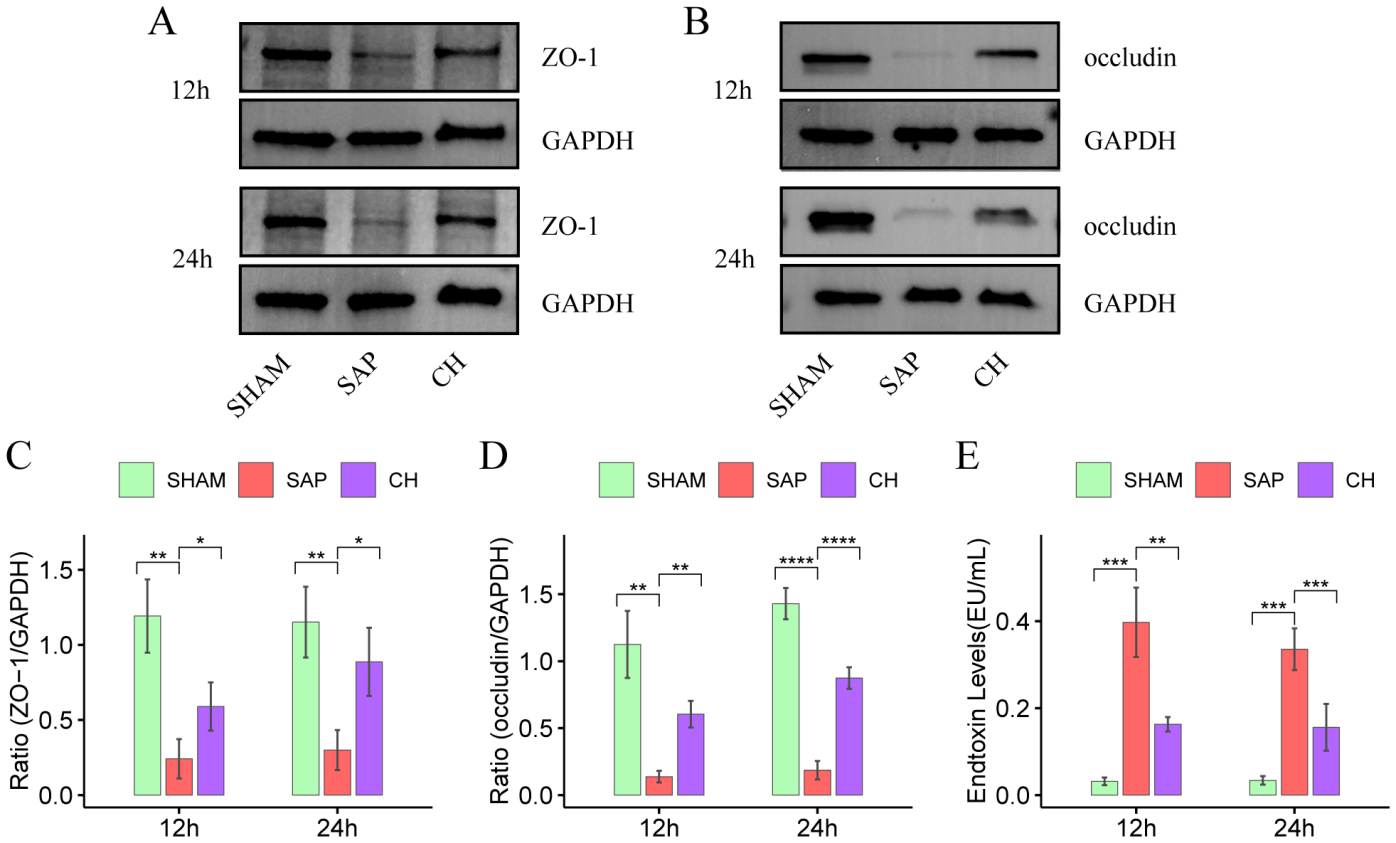
CH restored intestinal mucosal barrier function and reduced intestinal permeability in SAP rats. **(A)** Protein levels of ZO-1 by western blot. **(B)** Protein levels of occludin by western blot. GAPDH was used as the loading control. **(C)** Expression quantitation of ZO-1 relative to GAPDH in **(A)**. **(D)** Expression quantitation of occludin relative to GAPDH in **(B)**. **(E)** The level of serum endotoxin. For **(A-D)**, n = 4 and for **(E)**, n = 5. (*P < 0.05, **P < 0.01, ***P < 0.001, ****P < 0.0001, vs. SAP group).

The TJ proteins ZO-1 and occludin are key markers of intestinal epithelial integrity. The IHC results showed that in the same subgroups at 12h and 24h, the expression of both ZO-1 (**Figures 2C, D**) and occludin (**Figures 2E, F**) was significantly decreased in the SAP group compared to the SHAM group (P < 0.05). In contrast, the expression of both ZO-1 and occludin was significantly improved in the CH group compared to the SAP group (P < 0.05). Western blot analysis further confirmed that rats with SAP had downregulated the ZO-1 (**Figures 3A, C**) and occludin (**Figures 3B, D**) expression in the colonic tissue of compared to the SHAM group (P < 0.05). The levels of ZO-1 and occludin were increased in the CH group compared to the SAP group (P < 0.05). In addition, in the same subgroups at 12h and 24h, the endotoxin levels (**Figure 3E**) were significantly higher in the SAP group compared to the SHAM group (P < 0.05). In contrast, it was significantly lower in the CH group compared to the SAP group (P < 0.05). These results suggested that CH ameliorated the intestinal mucosal barrier damage in SAP by maintaining TJ protein expression and reducing intestinal permeability.

### 3.3 CH modulated gut microbiota diversity, structure and metabolic function in SAP rats

**Figure 4:**
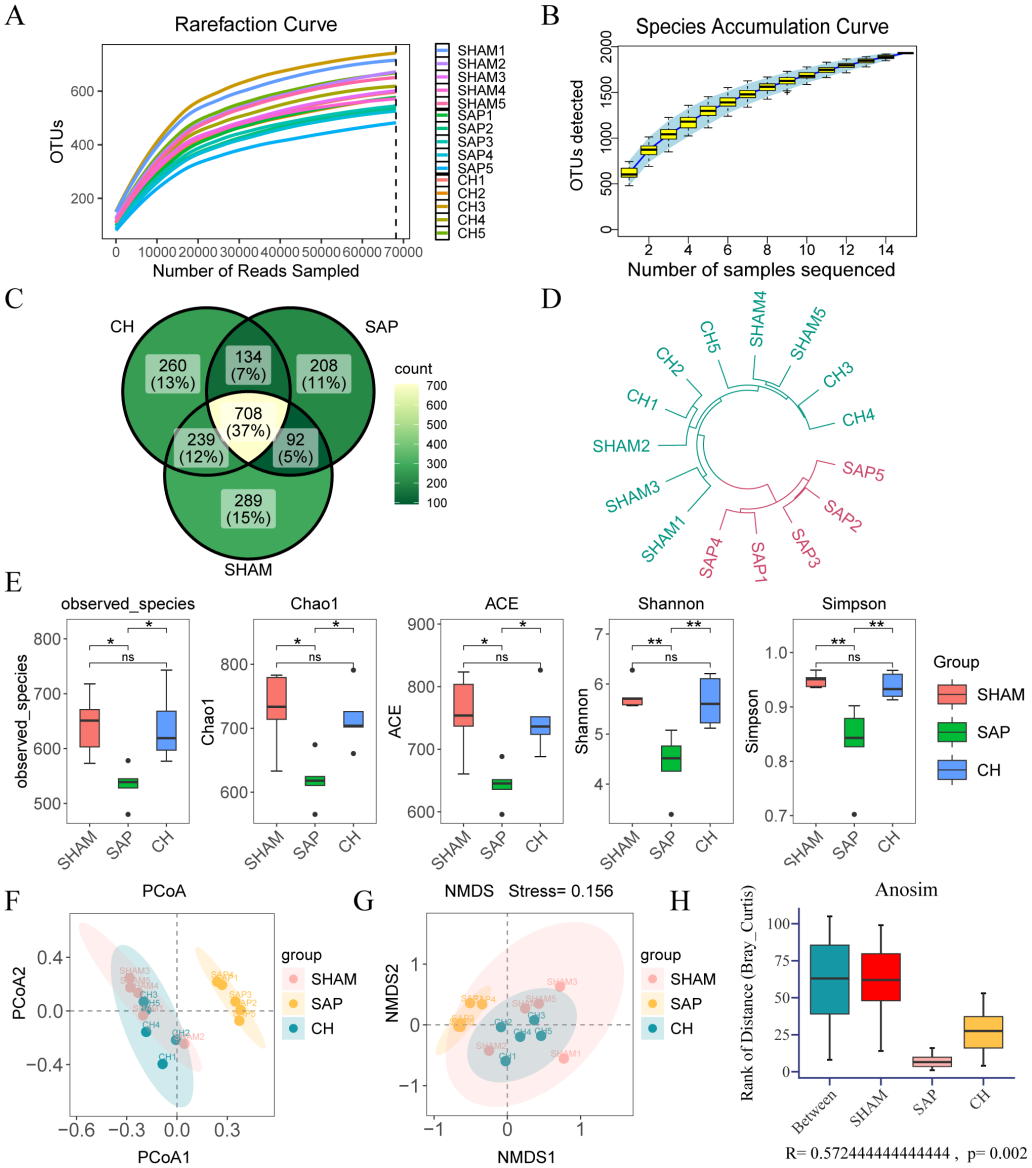
CH modulated the diversity and structure of the gut microbiota in SAP rats. **(A)** Rarefaction curve of OTUs. **(B)** Species accumulation curve of OTUs. **(C)** The Venn diagram analysis of OTUs. **(E)** The α diversity of the gut microbiota including observed-spacies, Chao1, ACE, Shannon and Simpson indices. The β diversity of the gut microbiota including **(F)** PCoA, **(G)** NMDS, **(H)** ANOSIM and **(D)** hierarchical clustering tree. (n = 5, ns, not significant, *P < 0.05, **P < 0.01).

In this study, a total of 1838753 original microbiota sequences were obtained by 16S rDNA sequencing. The sequences of the obtained samples were clustered and species annotated, and a total of 1942 OTUs were obtained, with 97% similarity between the samples. The rarefaction curve can reflect the rationality of sequencing samples, and the species accumulation curve represents the relationship between the sample capacity and the number of species. The two curves in this study tended to be flat, indicating that the amount of sequencing data was reasonable and could be further analyzed (**Figures 4A, B**). The Venn diagram (**Figure 4C**) reflects the differences between each group at the level of OTUs, with 289, 208, and 260 in the SHAM, SAP, and CH groups.

The observed-spacies, Chao1, ACE, Shannon, and Simpson indices were used to reflect the α diversity (Liu et al., 2019), which reflects species richness and evenness (**Figure 4E**). The richness and diversity of the gut microbiota were significantly lower in the SAP group compared to the SHAM group (P < 0.05), whereas they were significantly higher in the CH group compared to the SAP group (P < 0.05). In addition, β diversity was also evaluated by principal coordinate analysis (PCoA), non-metric multidimensional scaling (NMDS) analysis based on Bray-Curtis distances between microbial genera, analysis of similarities (ANOSIM), and hierarchical clustering tree, which showed that the gut microbiota was significantly clustered among the three groups at the OTUs level, with the distribution of the CH group closer to the SHAM group (**Figures 4D, F-H**).

**Figure 5:**
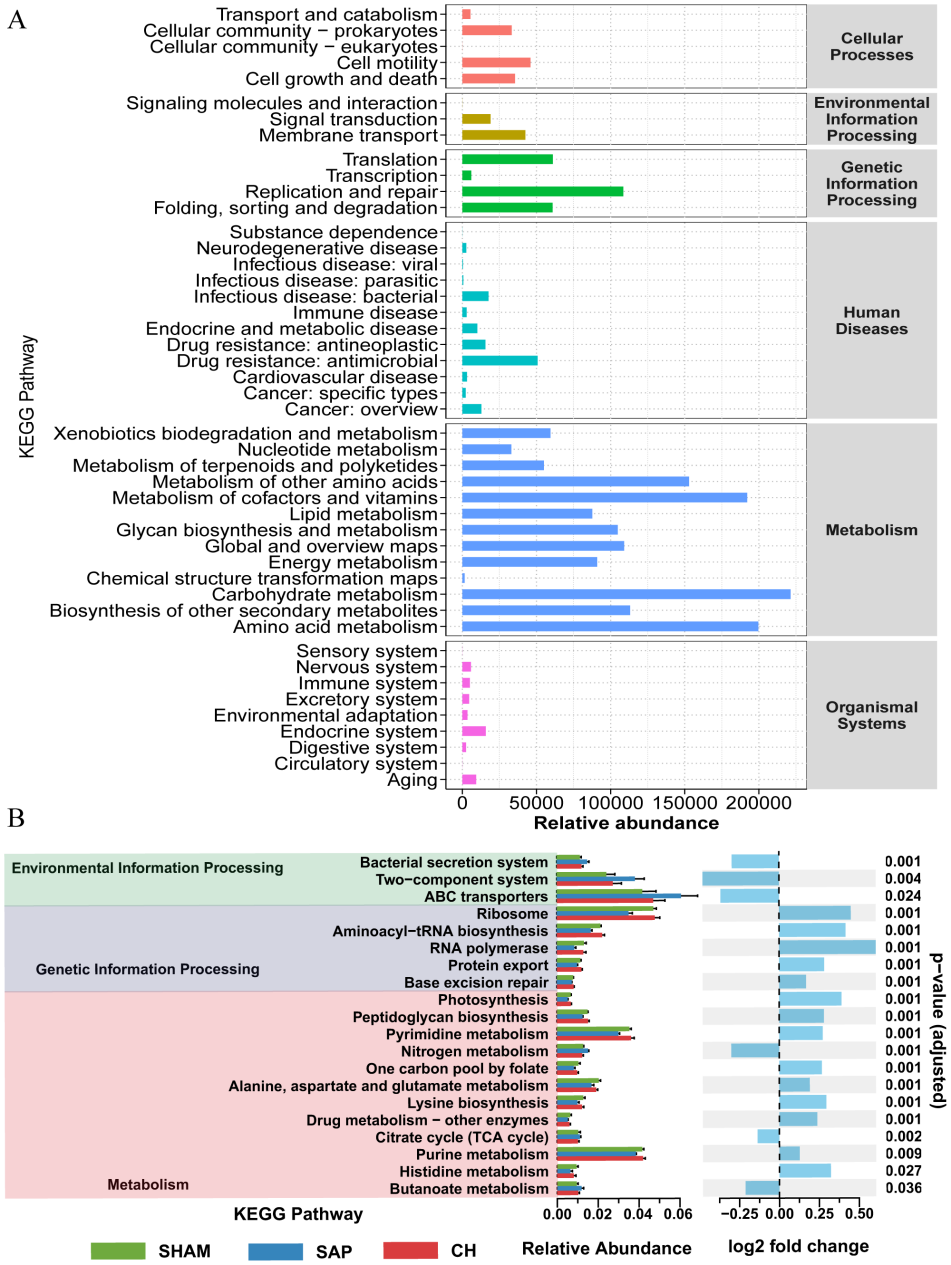
CH modulated the metabolic function of the gut microbiota in SAP rats. **(A)** Analysis of KEGG pathway enrichment in the gut microbiota. **(B)** Analysis of differences in KEGG metabolic pathways between groups.

The effect of CH on potential metabolic pathways in the gut microbiota of SAP rats was predicted using PICRUSt analysis based on 16S rDNA gene sequencing data and the KEGG database. The KEGG pathways of the three groups of rats were mainly involved in two major metabolic pathways, namely metabolism and genetic information processing. The functional categories were mainly related to carbohydrate metabolism, energy metabolism, lipid metabolism, etc. (**Figure 5A**). The differences in KEGG level 2 and level 3 within the KEGG level 1 metabolic categories between the groups (**Figure 5B**) showed that the differences in the metabolic functions of the gut microbiota of the rats in the SAP and SHAM groups were mainly related to amino acid metabolism (alanine, aspartate and glutamate metabolism, lysine biosynthesis, histidine metabolism), carbohydrate metabolism (butanoate metabolism), and so on. In contrast, CH intervention brought the predicted gut microbiota function of SAP rats closer to that of rats in the SHAM group. In summary, these results suggested that CH alleviated SAP-induced gut microbiota dysbiosis.

### 3.4 CH modulated the gut microbiota composition at different taxonomic levels in SAP rats

**Figure 6:**
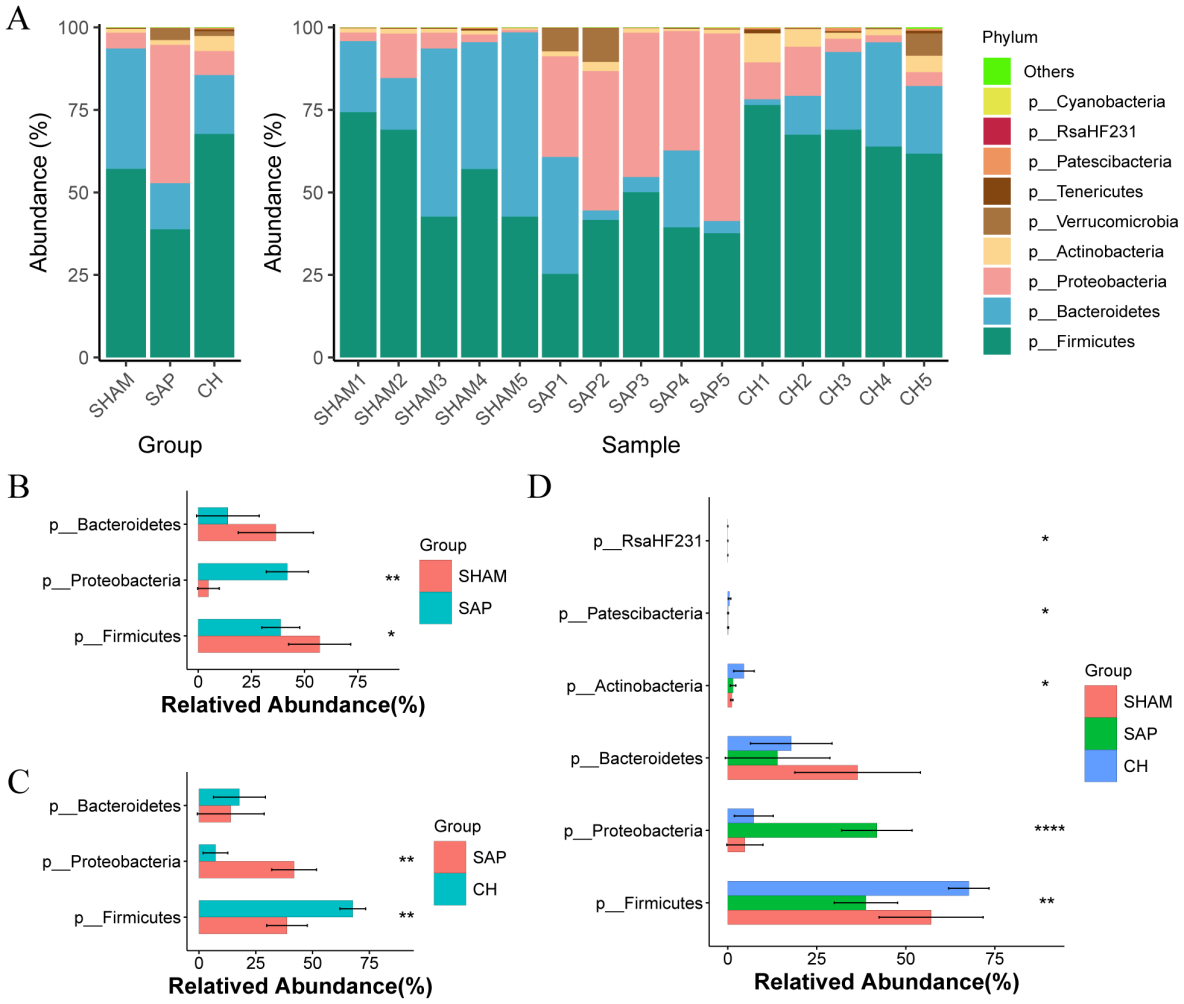
CH modulated gut microbiota composition at the phylum level in SAP rats. **(A)** Composition of the gut microbiota at the phylum level. **(B)** The representative differential phyla in the SHAM and SAP groups. **(C)** The representative differential phyla in the SAP and CH groups. **(D)** The representative differential phyla among three groups. **(B, C)** analyzed by Wilcoxon rank-sum test, **(D)** analyzed by ANOVA test. (n = 5, *P < 0.05, **P < 0.01, ****P < 0.0001).

At the phylum level, the three groups were mainly composed of Firmicutes, Bacteroidetes, and Proteobacteria, which accounted for 98.40%, 94.67%, and 92.82% of the gut microbiota in the SHAM, SAP, and CH groups, respectively (**Figure 6A**). Among them, the SHAM group was dominated by Firmicutes (57.12%) and Bacteroidetes (36.47%), which are important components for the production of SCFAs and maintenance of intestinal homeostasis. Compared to the SHAM group (**Figure 6B**), the percentage of Firmicutes (38.79%) in the SAP group was significantly decreased (P < 0.05), and the percentage of Proteobacteria (41.89%), which produce more pathogenic bacteria, was significantly higher (P < 0.05). However, CH treatment can significantly reverse these changes (p < 0.05; **Figure 6C**). In addition, the comparison of the three groups by ANOVA test showed differences in 5 phyla (**Figure 6D**).

**Figure 7:**
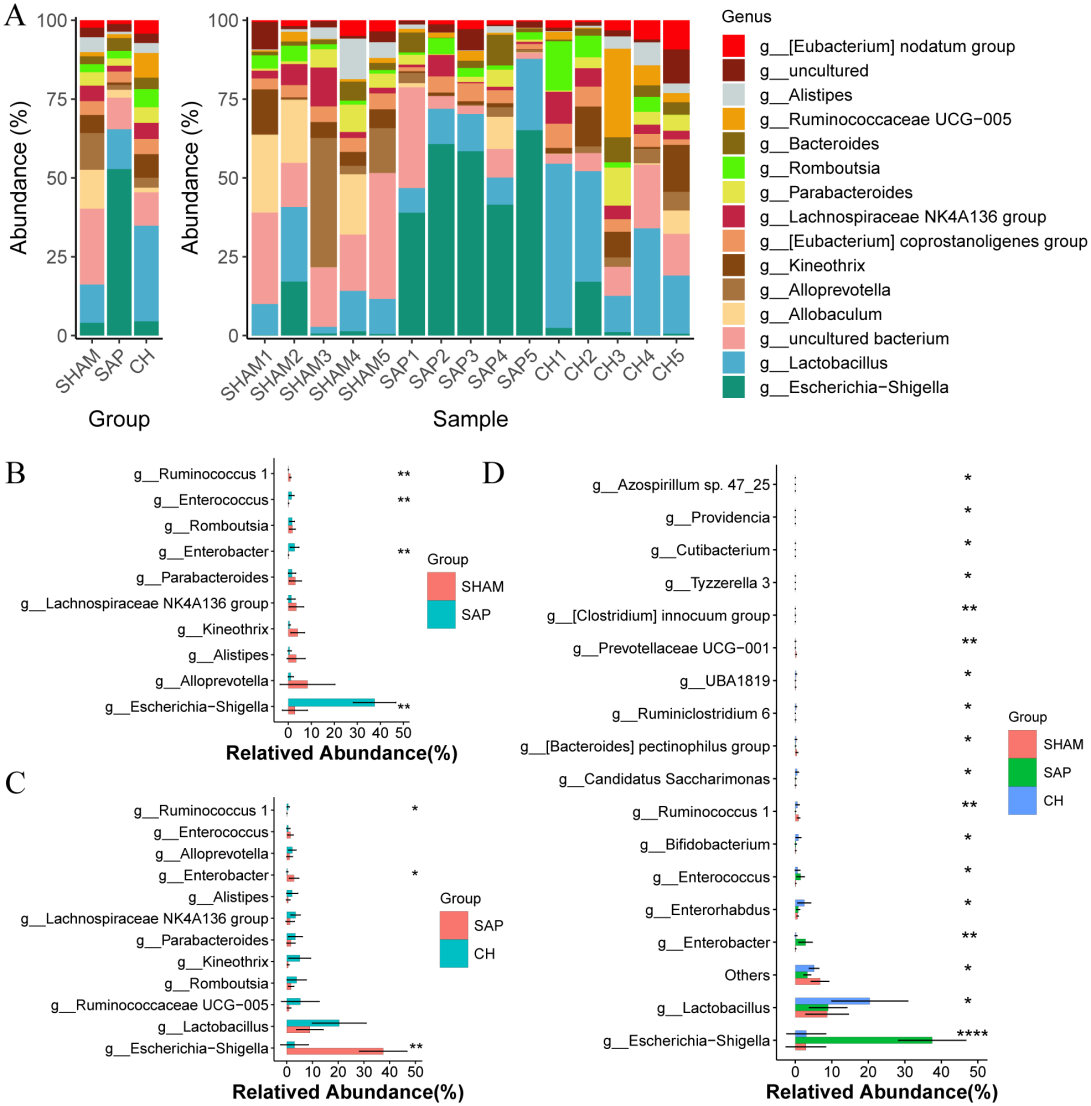
CH modulated gut microbiota composition at the genus level in SAP rats. **(A)** Composition of the gut microbiota at the genus level. **(B)** The representative differential genera in the SHAM and SAP groups. **(C)** The representative differential genera in the SAP and CH groups. **(D)** The representative differential genera among three groups. **(B, C)** analyzed by Wilcoxon rank-sum test, **(D)** analyzed by ANOVA test. (n = 5, *P < 0.05, **P < 0.01, ****P < 0.0001).

The composition of the gut microbiota changed significantly at the genus level (**Figure 7A**), where the bacterial genera of Ruminococcus 1, Parabacteroides, and Lachnospiraceae NK4A136 group were reduced in the SAP group compared to the SHAM group, and Escherichia-Shigella, Escherichia, Enterococcus, and Enterobacter were significantly increased (**Figure 7B**). Compared to the SAP group, and the CH group had an increased percentage of potentially beneficial bacteria such as Ruminococcus 1, Lachnospiraceae NK4A136 group, Parabacteroides, and Lactobacillus, which maintain intestinal homeostasis, promote the production of SCFAs, protect the intestinal mucosal barrier, and inhibit inflammation, and a decreased percentage of pathogenic bacteria Escherichia-Shigella, Enterococcus, and Enterobacter (**Figure 7C**). Furthermore, the comparison of the three groups by ANOVA test showed differences in 18 genera (**Figure 7D**).

**Figure 8:**
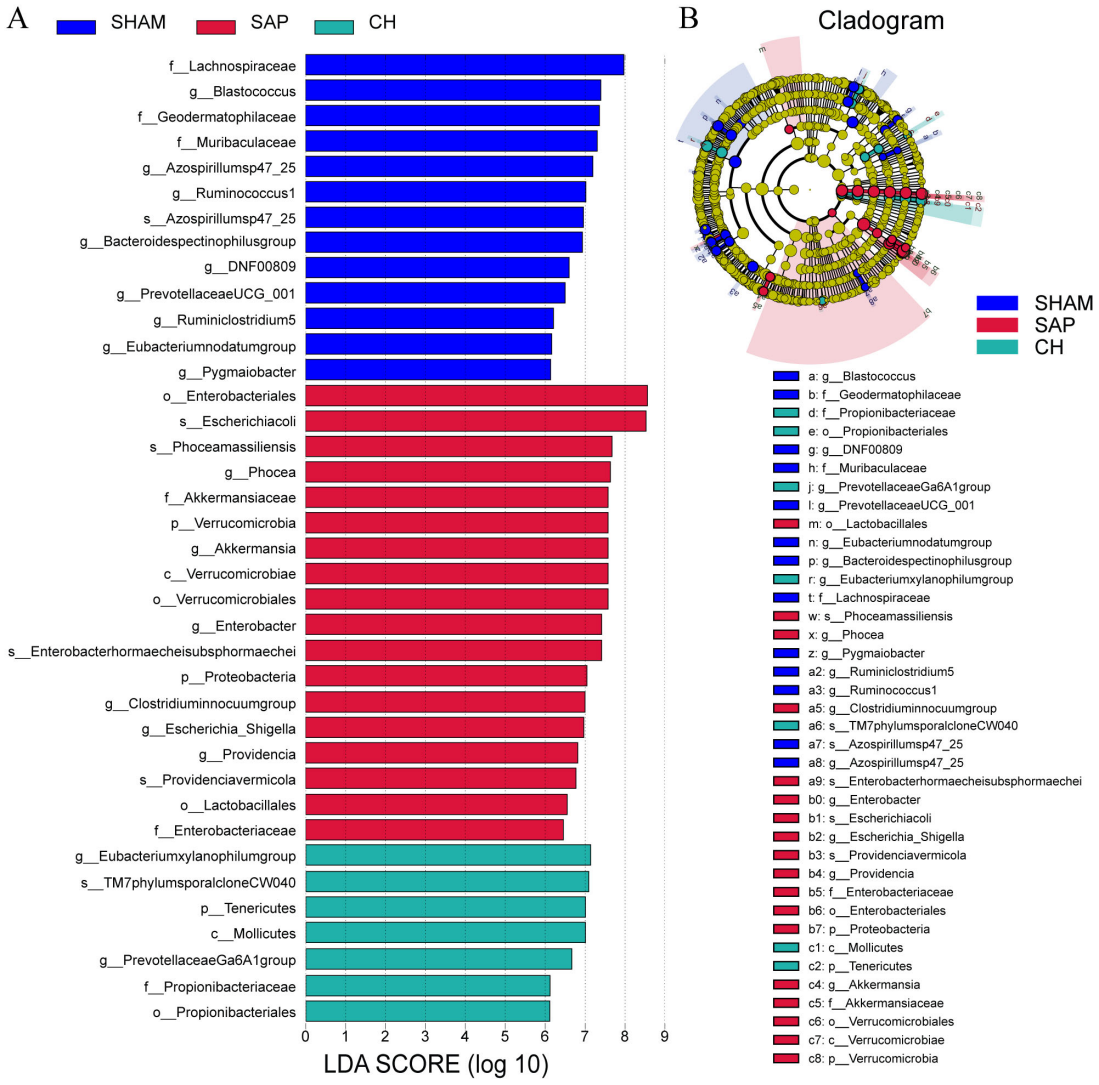
CH modulated the gut microbiota composition at different taxonomic levels in SAP rats. **(A)** LDA analysis. **(B)** LEfSe analysis. (LDA score ≥ 5.0).

In addition, LDA and LEfSe analyses were performed to identify taxonomic biomarkers in the gut microbiota. The LDA bar chart (**Figure 8A**) presented the key biomarkers for each group. At the same time, the cladogram (**Figure 8B**) illustrated the taxonomic hierarchy of these biomarkers, summarized from the phylum to the species level. 11, 6 and 5 biomarkers were identified in the SHAM, SAP and CH groups, respectively, including Prevotellaceae UCG-001, Lachnospiraceae, Ruminococcus 1 and Escherichia-Shigella. In brief, these obvious changes in the gut microbiota at different taxonomic levels under the intervention of CH confirmed the regulatory effect of CH on the gut microbiota.

### 3.5 CH improved intestinal mucosal barrier function in SAP rats by increasing the content of SCFAs

**Figure 9:**
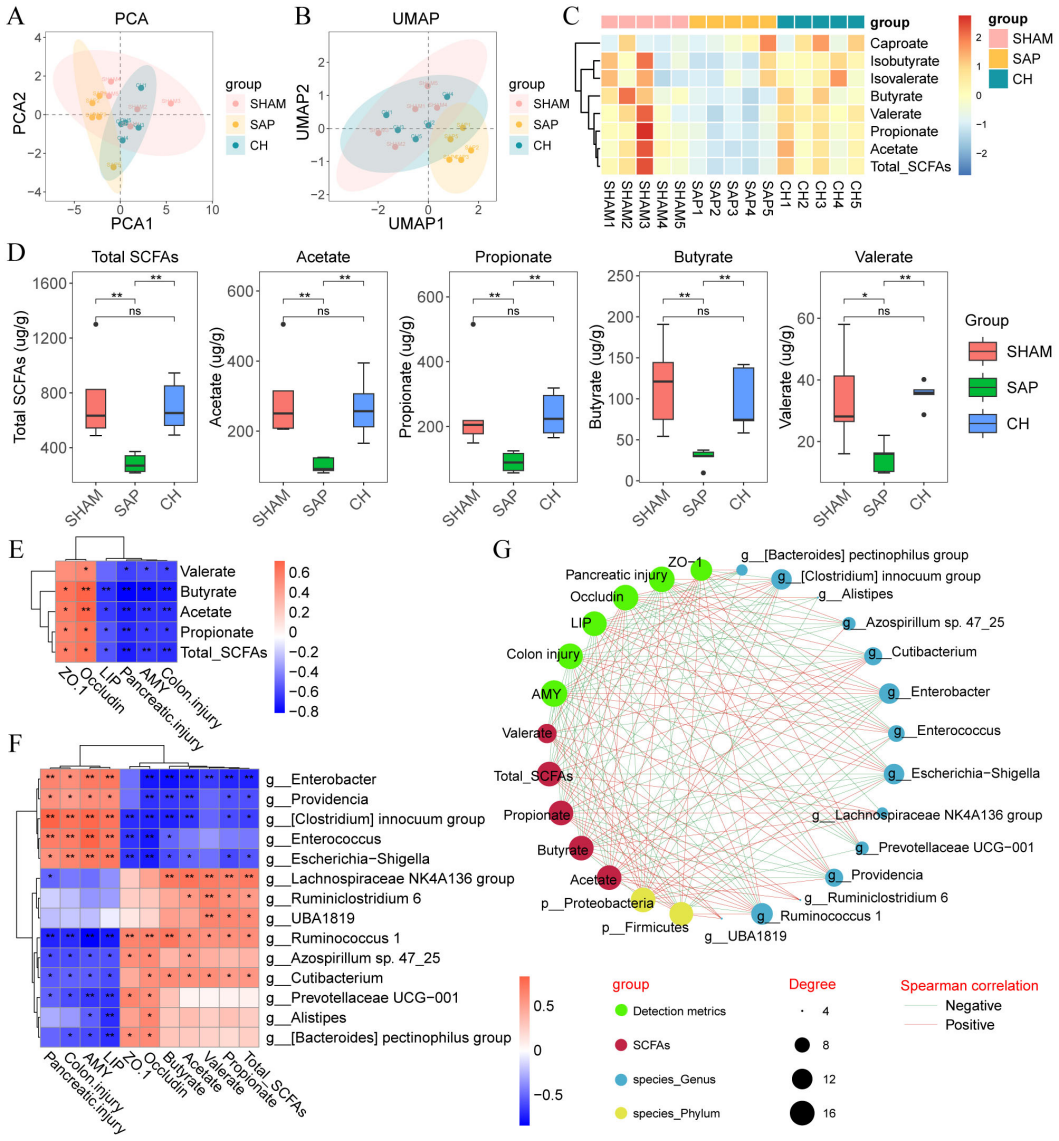
CH improved intestinal mucosal barrier function in SAP rats by increasing the levels of SCFAs. **(A)** PCA plot of SCFAs. **(B)** UMAP plot of SCFAs. **(C)** Heatmap of SCFAs. **(D)** The levels of SCFAs in the gut contents of each group. **(E)** Heatmap of correlation between SCFAs and disease phenotypes in SAP rats. **(F)** Heatmap of correlation between gut microbiota, SCFAs and disease phenotypes in SAP rats. **(G)** Integrative network between gut microbiota, SCFAs and disease phenotypes. Lines connecting nodes indicate positive (red) or negative (blue) correlations. **(D)** analyzed by Wilcoxon rank-sum test, **(E, F)** analyzed by Spearman correlation analysis. (n = 5, *P < 0.05, **P < 0.01).

SCFAs, as important metabolites of the gut microbiota, have a significant impact on intestinal homeostasis and immune regulation. The results of principal component analysis (PCA) and uniform manifold approximation and projection (UMAP) analyses (**Figures 9A, B**) suggested that the distribution of the CH group was close to that of the SHAM group, indicating the similarity of the composition of SCFAs between the SHAM and CH groups. Further analysis revealed (**Figures 9C, D**) that the levels of acetate, propionate, butyrate and valerate as well as total SCFAs were significantly decreased in the SAP group compared to the SHAM group (P < 0.05), whereas the intervention of CH restored them almost to the level of the SHAM group (P<0.05).

Spearman correlation analysis was used to determine the correlation between gut microbiota, SCFAs and disease phenotypes in SAP rats. Correlation analysis of SCFAs and phenotypes (**Figure 9E**) showed that the level of SCFAs was negatively correlated with pancreatic injury (amylase and lipase) and pathological damage (pancreatic and colonic pathological scores) and positively correlated with intestinal barrier proteins (ZO-1 and occludin). In addition, correlation analysis of gut microbiota with SCFAs showed that the relative abundance of Enterobacter, Enterococcus, and Escherichia-Shigella were negatively correlated with the level of SCFAs, and Lachnospiraceae NK4A136 group, Ruminococcus 1, and Prevotellaceae UCG-001 were positively correlated with the level of SCFAs (**Figure 9F**). Next, correlation analysis of gut microbiota and phenotypes showed that Enterobacter, Enterococcus and Escherichia-Shigella were positively correlated with pancreatic injury (amylase and lipase) and pathological damage (pancreatic and colonic pathological scores) and negatively correlated with intestinal barrier proteins (ZO-1 and occludin), while Lachnospiraceae NK4A136 group, Ruminococcus 1 and Prevotellaceae UCG-001 showed the exact opposite trend (**Figure 9F**). Finally, the associations between gut microbiota, SCFAs and disease phenotypes in SAP rats after CH intervention were visualized using correlation network plots (**Figure 9G**). These results suggested that CH treatment increased the relative abundance of SCFAs-producing bacteria in the rats and that SCFAs alleviated SAP by maintaining intestinal mucosal barrier function.

## 4 Discussion

Previous studies based on network pharmacology have identified a total of 168 active compounds in CH, including key compounds such as luteolin and quercetin (Yang et al., 2024a). Research suggested that luteolin restores the diversity and structure of the gut microbiota and maintains intestinal barrier function in ulcerative colitis rats by modulating genera such as Lactobacillus and Lachnospiraceae NK4A136 group (Li et al., 2021). Quercetin increases the relative abundance of Lactobacillus in the cecum and improves intestinal barrier function by modulating the gut microbiota composition and promoting TJ proteins expression (Uyanga et al., 2021). Lactobacillus is a probiotic that reduces intestinal inflammation and protects the intestinal barrier by promoting mucus production and barrier-related proteins and secreting antimicrobial substances (Dempsey and Corr, 2022). For example, a specific Lactobacillus strain, Lactobacillus rhamnosus GG (ATCC 53103) (L.GG), can increase the gene expression of ZO-1, claudin and occludin in Caco-2 cells (Orlando et al., 2014). Our results suggested that CH reduced colonic pathological damage in SAP rats, restored the expression of ZO-1 and occludin proteins, and meanwhile, CH increased the abundance of beneficial bacteria, such as Lactobacillus and Lachnospiraceae NK4A136 group, which significantly alleviated the dysbiosis of the gut microbiota in SAP rats, and the diversity and structure of the gut microbiota were restored. Taken together, the herbs in CH exerted a wide range of regulatory effects on the gut microbiota and mechanical barriers.

Bacterial and endotoxin translocation due to damage to the intestinal barrier, triggering an inflammatory cascade, is a “second hit” against SAP (Su and Tang, 2021). Endotoxin is a toxic bacterial component produced by Gram-negative bacteria that induces a strong inflammatory response in mammals (Brown and Heneka, 2024), mainly by binding to the proteins Toll-like receptor 4 (TLR4) and myeloid differentiation factor 2 (MD2), which activates pro-inflammatory pathways leading to the production of large amounts of pro-inflammatory cytokines such as IL-6, IL-1β and TNF-α (Bryant et al., 2010). Escherichia-Shigella is an opportunistic Gram-negative bacterial pathogen that invades the colonic and rectal mucosa, causing a strong inflammatory response, and Enterobacter provokes SIRS and intestinal bacterial translocation (Wu et al., 2023). In this study, CH was able to reduce serum amylase and lipase levels and attenuate pancreatic pathological damage in SAP rats, which is consistent with our previous findings (Yang et al., 2024a; Liu et al., 2025). In addition, CH inhibited endotoxin levels and reduced the abundance of pathogenic bacteria such as Escherichia-Shigella and Enterobacter in SAP rats. Previous *in vivo* and *in vitro* experiments (Fu et al., 2024; Yang et al., 2024a; Liu et al., 2025) showed that serum levels of TNF-α, IL-1β and IL-6 were significantly increased in the AP model group compared to the control group, the same levels were observed in pancreatic follicular cells of AR42J rats, and IL-1β levels were also significantly increased in the intestine of SAP rats. In contrast, CH administration significantly reduced the levels of these inflammatory factors. In conclusion, CH played a protective role in SAP by modulating gut microbiota composition, inhibiting systemic inflammatory responses and reducing bacterial and endotoxin translocation.

SCFAs are important metabolites produced by the fermentation of dietary fiber by the gut microbiota and have a wide range of biological functions, including maintaining intestinal homeostasis, regulating host immune responses and repairing the intestinal mucosal barrier (Sun et al., 2018; Yang et al., 2020; Xiong et al., 2022). Butyrate, one of the SCFAs, has been shown to attenuate AP by ameliorating intestinal barrier dysfunction (Xia et al., 2023), and the mechanism may be related to the inhibitory effect of immune-activated histone deacetylase (HDAC) (Pan et al., 2019; Korsten et al., 2023). The gut microbiota that produce SCFAs are usually commensal probiotics such as Parabacteroides, Ruminococcus 1, Prevotellaceae UCG-001, Lachnospiraceae NK4A136 group, etc., which are capable of exerting positive effects on the intestinal barrier (Fusco et al., 2023; Wen et al., 2023; Zhang et al., 2023). Parabacteroides can produce acetate, which alleviates heparinase-exacerbated AP by reducing neutrophil infiltration (Lei et al., 2021). Ruminococcus 1 regulates the production of SCFAs through saccharolytic capacity, thereby improving colonic health (Xie et al., 2019). Prevotellaceae UCG-001 inhibits intestinal inflammation and improves intestinal mucosal barrier function by producing butyrate (Wang et al., 2021). Consistently, our study indicated that the relative abundance of a variety of probiotic bacteria, represented by the Lachnospiraceae NK4A136 group, was positively correlated with SCFAs levels. In addition, SCFAs-producing bacteria represented by Ruminococcus 1 were positively correlated with intestinal barrier proteins (ZO-1 and occludin) and negatively correlated with intestinal damage (colonic pathological scores) and pancreatic damage (amylase, lipase and pancreatic pathological scores). In conclusion, CH exerted the protective effects of SAP by increasing the number of SCFAs-producing bacteria to maintain the function of the intestinal mucosal barrier.

Furthermore, there are some limitations to this study. Firstly, this study only investigated the mechanism of CH in the treatment of SAP in terms of microbial homeostasis, but the underlying molecular mechanisms were not investigated. Secondly, this study lacks analysis of metabolites other than SCFAs. To remedy these deficiencies, we will further integrate transcriptomics and non-targeted metabolomics to reveal the specific targets and comprehensive mapping of metabolites of CH to ameliorate SAP. In addition, collecting and collating clinical information on CH for SAP through large-scale clinical trials is a key part of advancing the development of new drugs for the treatment of SAP.

## 5 Conclusion

**Figure 10:**
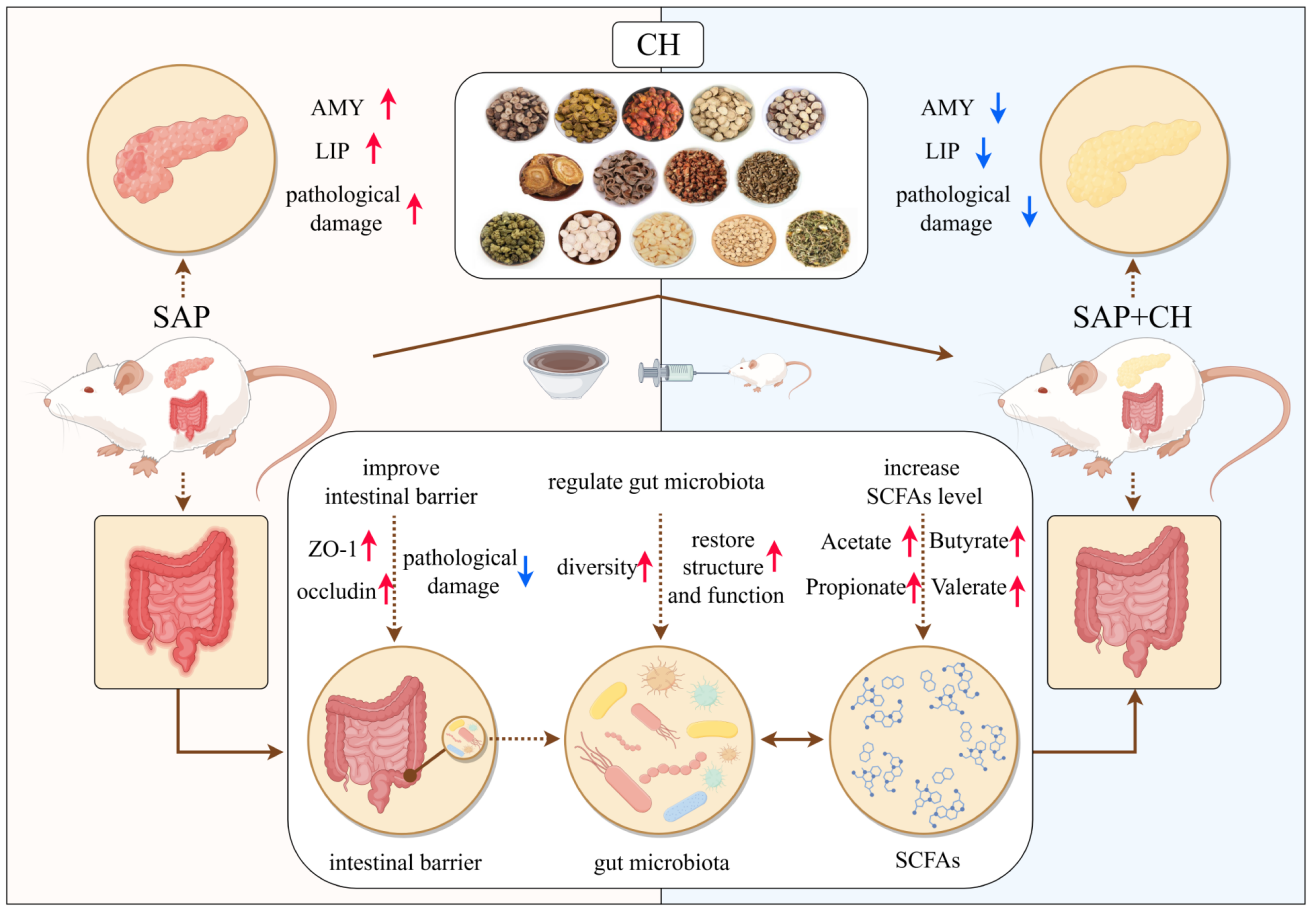
Schematic diagram of the possible mechanisms of CH to ameliorate SAP symptoms. The figure was drawn by Figdraw.

In conclusion, this study showed that by targeting the negative feedback pathway of gut microbiota dysfunction and intestinal mucosal barrier injury during SAP, CH could exert a protective effect against SAP by regulating gut microbiota homeostasis, promoting the expression of SCFAs and intestinal mucosal barrier repair (**Figure 10**). All the results suggested that CH may be one of the potential therapeutic agents to ameliorate SAP-associated acute intestinal injury and gut microbiota dysfunction.

## Data Availability

The original contributions presented in the study are publicly available. This data can be found here: https://www.ncbi.nlm.nih.gov/sra/PRJNA1220697, PRJNA1220697.

## Glossary

ANOSIM: analysis of similarities
AP: Acute pancreatitis
BCA: Bicinchoninic Acid Assay
CH: Chaihuang Qingyi Huoxue Granule
DAB: 3,3'-diaminobenzidine
ECL: Electrochemiluminescence
GC-MS: gas chromatography-mass spectrometry
HDAC: histone deacetylase
HE: hematoxylin-eosin
HRP: horseradish peroxidase
IECs: intestinal epithelial cells
IHC: Immunohistochemistry
IL-1β: interleukin-1β
IL-6: interleukin-6
KEGG: Kyoto Encyclopedia of Genes and Genomes
L.GG: Lactobacillus rhamnosus GG
LDA: Linear discriminant analysis
LEfSe: LDA effect size
MD2: myeloid differentiation factor 2
MODS: multiple organ dysfunction syndrome
NMDS: non-metric multidimensional scaling
OTUs: operational taxonomic units
PCA: principal component analysis
PCoA: principal coordinate analysis
PICRUSt: phylogenetic investigation of communities using reconstruction of unobserved states
SAP: severe acute pancreatitis
SCFAs: Short-chain fatty acids
SEM: standard error of mean
SIRS: systemic inflammatory response syndrome
TCM: traditional Chinese medicine
TJs: tight junctions
TLR4: Toll-like receptor 4
TNF-α: tumor necrosis factor-α
UMAP: uniform manifold approximation and projection
ZO-1: zona occludens-1.
